# Differences in temperature-sensitive expression of PcG-regulated genes among natural populations of *Drosophila melanogaster*

**DOI:** 10.1093/g3journal/jkab237

**Published:** 2021-07-14

**Authors:** Susanne Voigt, Luise Kost

**Affiliations:** Applied Zoology, Faculty of Biology, Technische Universität Dresden, Dresden 01217, Germany

**Keywords:** *Drosophila melanogaster*, evolution of gene regulation, Polycomb group, environmental sensitivity, expression plasticity, temperature-dependent expression

## Abstract

Environmental temperature can affect chromatin-based gene regulation, in particular in ectotherms such as insects. Genes regulated by the Polycomb group (PcG) vary in their transcriptional output in response to changes in temperature. Expression of PcG-regulated genes typically increases with decreasing temperatures. Here, we examined variations in temperature-sensitive expression of PcG target genes in natural populations from different climates of *Drosophila melanogaster*, and differences thereof across different fly stages and tissues. Temperature-induced expression plasticity was found to be stage- and sex-specific with differences in the specificity between the examined PcG target genes. Some tissues and stages, however, showed a higher number of PcG target genes with temperature-sensitive expression than others. Overall, we found higher levels of temperature-induced expression plasticity in African tropical flies from the ancestral species range than in flies from temperate Europe. We also observed differences between temperate flies, however, with more reduction of expression plasticity in warm-temperate than in cold-temperate populations. Although in general, temperature-sensitive expression appeared to be detrimental in temperate climates, there were also cases in which plasticity was increased in temperate flies, as well as no changes in expression plasticity between flies from different climates.

## Introduction

The colonization of new environments requires species to adapt to a range of novel abiotic and biotic conditions. Environmental temperature is a critical factor in determining species abundance and geographic distribution, in particular for ectotherms such as insects (Andrewartha and Birch 1954; Cossins 2012). The fruit fly *Drosophila melanogaster*, as a cosmopolitan species, has adapted to a wide range of thermal environments (Hoffmann *et al.* 2003; Ayrinhac *et al.* 2004; Pool *et al.* 2017). From its tropical origins in southern-central Africa, it has spread to nearly all regions around the world including temperate regions of Europe (David and Capy 1988; Lachaise and Silvain 2004; Stephan and Li 2007; Laurent *et al.* 2011; Pool *et al.* 2012; Arguello *et al.* 2019; Kapopoulou *et al.* 2020).

Temperate climates are characterized by low and fluctuating temperature. Fluctuations in temperatures can affect chromatin-based gene regulation (Fauvarque and Dura 1993). The evolutionary conserved Polycomb group (PcG) of proteins consists of important epigenetic regulators in *Drosophila* (Kassis and Brown 2013; Simon and Kingston 2013; Steffen and Ringrose 2014; Entrevan *et al.* 2016; Giner-Laguarda and Vidal 2020; Kuroda *et al.* 2020) that appear to be sensitive to temperature.

PcG-regulated genes vary in their transcriptional output in response to changes in temperature at which flies are reared or held. Expression of PcG-regulated genes typically increases with decreasing temperature (Fauvarque and Dura 1993; Chan *et al.* 1994; Zink and Paro 1995; Bantignies *et al.* 2003; Gibert *et al.* 2011; Voigt *et al.* 2015). Classically, this has been shown in transgenic assays in which reporter gene expression is controlled by PcG-regulatory sequences derived from prominent PcG target genes (Fauvarque and Dura 1993; Chan *et al.* 1994; Zink and Paro 1995; Bantignies *et al.* 2003; Gibert *et al.* 2011). The *miniwhite* reporter for red eye color was often used to demonstrate temperature-sensitive expression of genes regulated by the PcG (Fauvarque and Dura 1993; Chan *et al.* 1994; Zink and Paro 1995; Bantignies *et al.* 2003). Although there is some evidence that the expression of PcG genes can also vary with temperatures (Voigt *et al.* 2015; Zhao *et al.* 2015), the nature of how temperature changes affect PcG-mediated gene regulation is still largely unknown. PcG proteins work in multi-protein complexes that create large repressive domains at their target sites which are characterized by trimethylation of histone 3 at lysine 27 (H3K27me3) (Kassis and Brown 2013; Simon and Kingston 2013; Steffen and Ringrose 2014; Entrevan *et al.* 2016; Giner-Laguarda and Vidal 2020; Kuroda *et al.* 2020). PcG target genes are also regulated by another group of epigenetic regulators, the Trithorax group (TrxG). PcG and TrxG proteins function in an antagonistic manner to maintain repressed and activated transcription states, respectively (Steffen and Ringrose 2014; Kuroda *et al.* 2020). More recent evidence, however, suggests that PcG proteins not only maintain transcriptional repression of their targets but also modulate transcription levels by dampening expression levels of their transcriptionally active target genes (Enderle *et al.* 2011; Loubiere *et al.* 2016; Pherson *et al.* 2017).

Many of the target genes encode transcription factors with important roles in development and cell-fate speciation, including most prominently the HOX genes (Schuettengruber *et al.* 2009; Schwartz *et al.* 2010). Besides their role as developmental regulators, PcG proteins appear to be involved in the dynamic regulation of a wide variety of processes including cell cycle control, spermatogenesis, metabolism, cellular senescence, tissue homeostasis, mitochondrial function, and redox homeostasis (Schuettengruber *et al.* 2009; Pospisilik *et al.* 2010; Vidal 2014; Ugrankar *et al.* 2015).

Temperature-induced expression plasticity of PcG-regulated genes might have played a role while adapting to temperate climates. It could have been detrimental by shifting the transcriptional output from an optimum, reflecting an inability to buffer against fluctuations in temperature and to maintain consistent expression levels across temperatures. However, temperature-induced expression plasticity might have also been advantageous when changed expression levels contributed to phenotypic plastic responses that allowed the fly to produce phenotypes best suited for the respective environmental temperature. Finally, a plastic change in expression due to temperature might have also been neutral (or nearly neutral) with little effect on fitness (Levine and Begun 2008; Huang and Agrawal 2016; Mallard *et al.* 2020).

Temperature-sensitive gene expression has been comprehensively demonstrated in adult flies using whole-fly samples of one sex (Levine *et al.* 2011; Zhao *et al.*^2015^; Chen *et al.* 2015a,b). Less is known, however, about possible differences in temperature-induced expression plasticity between different sexes, fly stages and tissues, and changes thereof between flies from different climates. This study, therefore, tried to investigate changes in temperature-induced expression plasticity of PcG target genes in populations from different climates, as well as differences in expression plasticity across different fly stages, sexes, and tissues. We found temperature-induced expression plasticity to be stage-, sex-, and tissue-specific with differences in the specificity between the examined PcG target genes and populations.

## Materials and methods

### Fly lines and sample collection

Each of the three population samples consisted of eight isofemale, inbred lines derived from natural populations from Sweden (Kapopoulou *et al.* 2020), France and Zambia (Pool *et al.* 2012; Lack *et al.* 2016) (Table 1). All three populations are well-studied and eight lines from each were randomly chosen for this study. All lines were reared in separate vials, on standard cornmeal-sucrose-yeast medium with a light: dark cycle of 12:12 h. In order to control for generational effects, parent flies of the experimental generation were reared at 21°C and were transferred after hatching to the experimental temperatures of 15°C and 28°C to mate and oviposit. The resulting offspring was then reared at the respective temperature under density-controlled conditions (50 larvae per vial). Samples were collected from three different stages/sexes and three different types of tissue. The former included 5-day old mated adult flies (males and females, separately) and wandering third instar larvae (wL3), and the latter testis, ovaries, and female midguts dissected from adult flies. Adult and larval whole-fly samples from each line were collected and flash-frozen in liquid nitrogen and stored at −80°C. For each temperature and population, whole-fly samples and dissected tissues were evenly pooled from the eight lines of each population sample. Whole-fly samples consisted of pools of two individuals per line and four individuals per line were dissected for each type of tissue. Dissections were done in Shields and Sang M3 insect medium (Sigma-Aldrich). Dissected tissues were then immediately transferred into RNAlater (Qiagen).

**Table 1:** Origins of population samples and climates of origins

Population sample (collection site)	Mean annual temperature (°C)	Thermal range^*a*^ (°C)	Climate
Umeå, Sweden	2.7	−9.7 to 15.9	Cold-temperate
Lyon, France	11.6	2.6–21.0	Warm-temperate
Siavonga, Zambia	25.2	20.9–30.4	Tropical

^a^Thermal range corresponds to the minimum to maximum of mean monthly temperature throughout the year.

Climate data are based on the climate reference period from 1982 to 2012 (*en.climate-data.org*).

### Expression analysis

RNA was extracted using the MasterPure™ Complete DNA and RNA Purification Kit (Lucigen) and reverse-transcribed into cDNA using random primers and the High-Capacity cDNA Reverse Transcription Kit (Thermo Fisher Scientific). RNA purity was assessed using the ratio of absorbances at 260 and 280 nm (A260/A280 > 1.8). RT-qPCR reactions were performed with PowerUp™ SYBR™ Green Master Mix (Thermo Fisher Scientific) on a QuantStudio 5 cycler (Thermo Fisher Scientific). Primer was designed using QuantPrime (Arvidsson *et al.* 2008). Three biological replicates per population sample, rearing temperature and stage/sex/tissue were run in triplicates. Primer specificity was confirmed by melting curve analysis. No template controls (NTCs) and no reverse-transcription controls (NRTs) were included as negative controls to exclude contamination. Inter-run calibrated normalized relative expression was calculated using the qBase relative quantification framework (Hellemans *et al.* 2007). Both reference genes (*alphaTub84B* and *eIF-1A*) used for normalization were stably expressed across all samples, which was assessed by calculating the coefficient of variation and the M stability parameter as described in Hellemans *et al.* (2007).

### Statistical analysis

All statistical analyses were done on log-transformed inter-run calibrated normalized expression values. Mixed linear models were employed on both data sets, whole-fly and tissue-specific, to evaluate the overall effects of rearing temperature and origin on gene expression. All models included rearing temperature, the population as well as their interaction as fixed effects factors, and gene-specific expression as a random-effects factor. Depending on the model, stage/sex, tissue, PcG target/nontarget control, and general expression level were also considered as fixed factors. Categories of general expression levels of each tissue and stage/sex were derived from FlyBase (Graveley *et al.* 2011; Brown *et al.* 2014; Larkin *et al.* 2021). The significance of fixed effects was assessed with type III sums-of-squares tested with analyses of deviance based on the chi-square distribution as implemented in the R package car (Fox and Weisberg 2019). Starting with full models, best-fitting models were identified via stepwise model reduction based on the Akaike’s information criterion (AIC) by sequentially removing nonsignificant interaction terms. Simple linear modeling followed by ANOVA was used to evaluate the effects of rearing temperature, population, and their interaction on expression in each sex/stage and tissue and for each gene separately. Since the interaction between rearing temperature and the population was of particular interest, no model reduction was performed for the gene-specific models. *Post-hoc* analyses were done using Tukey HSD tests.

## Results

The aim of the study was to explore temperature-sensitivity of PcG regulation in order to assess whether there are differences in temperature-induced plasticity of PcG target genes among different stages, sexes, and tissues; as well as differences thereof between flies adapted to different climates. The latter included samples from three different populations: a cold-temperate from Sweden, a warm-temperate from France and a tropical from the species’ ancestral range in Zambia (Table 1). Because we were interested in aspects of temperature-sensitive expression of PcG target genes, we chose genes for which temperature-sensitive expression had already been shown for males in a slightly different temperature regimen (Zhao *et al.* 2015). In addition, we also included PcG target genes for which no temperature-sensitive expression was observed in the former study (*hh* and *Glut3*), as well as genes whose expression plasticity was reversed in respect to the temperature-induced expression plasticity typically associated with PcG regulation (*twi* and *HGTX*) (Supplementary Table S1).

**Figure 1:**
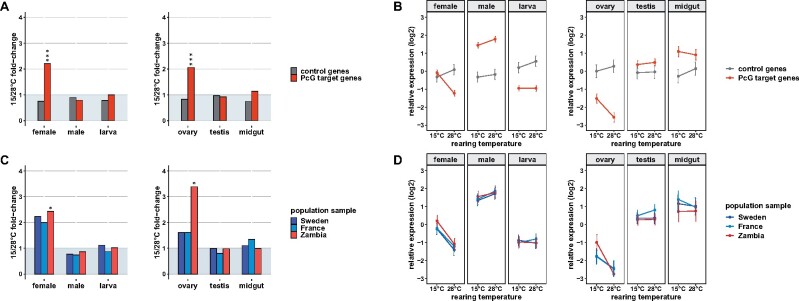
Gene expression in different stages/sexes and tissues at two different rearing temperatures. Variation in expression was analyzed using mixed linear models. Model-based means across PcG target or nontarget control genes are shown. (A) Fold-change in expression between 15°C and 28°C and (B) mean (±SE) expression at 15 and 28°C of PcG target and nontarget control genes. (C) Fold-change in expression between 15°C and 28°C and (D) mean (±SE) expression at 15°C and 28°C of PcG target genes in three different population samples. Significance levels were derived from *post-hoc* analyses using Tukey HSD tests (***P < 0.001, *P < 0.05).

**Figure 2:**
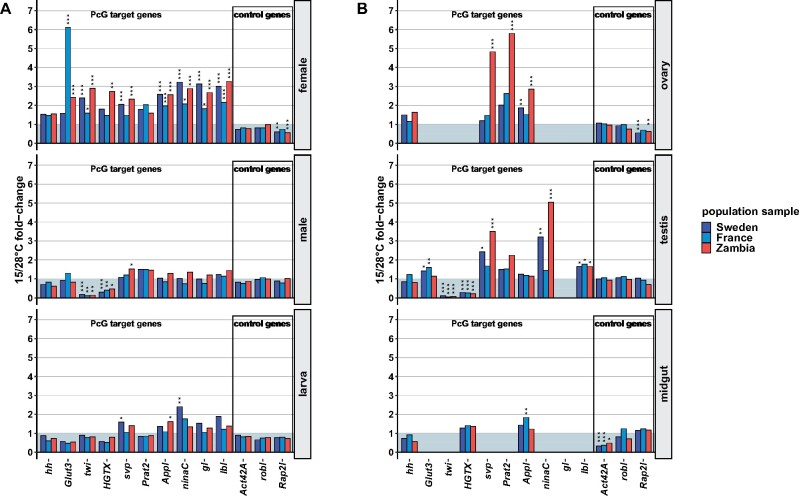
Changes in gene expression between rearing temperatures for each PcG target and nontarget control gene. Fold-changes between 15°C relative to 28°C are given for each (A) stage/sex and (B) tissue in each of the three population samples. Significance levels were derived from *post-hoc* analyses using Tukey HSD tests (***P < 0.001, **P < 0.01, *P < 0.05).

Expression plasticity as typically observed for PcG-regulated genes with increased transcription at lower temperature was observed for all examined PcG targets, except for *hh*, in at least one sex, stage, or tissue (Figure 2). PcG-type expression plasticity was not observed for control genes that are not regulated by the PcG and whose expression was also assessed in all stages, sexes, and tissues (Figures 1, A and B and 2, Supplementary Table S2). Temperature sensitivity of PcG target gene expression was also independent of expression level and observed for both low and high expression states (Supplementary Figure S1 and Table S4).

**Figure 3:**
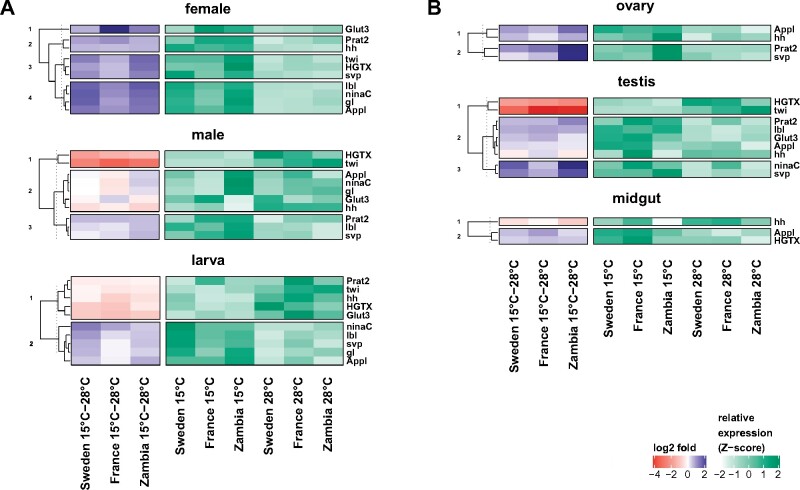
Cluster analysis of PcG target gene expression for each (A) stage/sex and (B) tissue. PcG target genes were clustered (k-means) based on their log2 fold-changes between the two rearing temperatures in the three population samples. Overexpression at 15°C is indicated by blue shades and overexpression at 28°C by red shades. Standardized relative expression levels (Z-score) for each rearing temperature and each population are shown as well.

As expected, PcG targets with increased male expression at higher temperatures in a previous study (Zhao *et al.* 2015), were also upregulated at 28°C compared to 15°C in males (*twi* and *HGTX*). In females, interestingly, the temperature effect on the expression of these two genes was reversed with higher expression at 15°C (Figures 2 and 3, Supplementary Figures S4 and S5, Tables S9 and S10). Otherwise, PcG target expression in males as well as in larvae was less affected by temperature compared to females, in which the majority of the studied PcG targets exhibited temperature-sensitivity characteristics for PcG regulation (Figures 1 and 2, Supplementary Tables S3 and S5).

Since whole-fly expression averages expression across the many tissues of the organism and the response to temperature of PcG target gene expression appear to differ between tissues (Voigt *et al.* 2015, 2019), we also monitored tissue-specific expression. For this, we chose three different adult tissues, as we were interested in temperature-sensitive expression and most of it was observed in adults. Females and males differed in the amount of temperature-sensitive expression with a higher amount in females, we, therefore, chose two female tissues (midgut, ovary) and one male tissue (testis) for the tissue-specific expression analysis. We included the reproductive organs of both sexes as these are likely the tissues with the strongest differentiation between the sexes. Not all of the studied PcG target genes were expressed in each tissue. Gene expression was detected only for subsets of the studied PcG targets which also differed between the tissues (Figure 2). Therefore, temperature-sensitive expression found in whole flies, in particular in females, derives unlikely only from the tissues examined here, instead of other types of tissues also seem to contribute to the observed temperature sensitivity in adults. Increased expression of *Glut3* and *twi* at 28°C relative to 15°C in males was, however, also found in testes (Figures 2 and 3, Supplementary Figures S3 and S4, Tables S5, S6, S8, and S9). Although PcG-type expression plasticity due to temperature was rare in whole-fly males for the considered PcG targets, five of them showed this type of temperature-sensitive expression with higher expression at the lower temperature in testes (Figures 2 and 3, Supplementary Figure S3, S6, S7, S9, and S11, Tables S6, S8, S11, S12, S14, and S16). Only three of the studied PcG targets were expressed in female midguts and only one of them was seemingly affected by temperature in its expression (Figure 2, Supplementary Figure S8, Table S6 and S13). Female midguts appear therefore not to contribute much to the observed temperature-sensitive expression in females.

The plastic response of PcG target gene expression to rearing temperature varied between flies from different climates (Figures 1, C and D and 2, Supplementary Tables S5 and S6). Overall, temperature-induced expression plasticity with increased transcription at lower temperatures as it is characteristic for PcG regulation was less pronounced in temperate than in ancestral tropical fly populations. PcG targets that exhibited PcG-type temperature-sensitive expression in a specific stage/sex or tissue, in general, showed a greater degree thereof in tropical than in temperate flies (Figures 1, C and D and 2). Interestingly, differences in this buffering effect were also obvious between temperate populations with reduction in temperature-sensitive expression occurring more often in warm-temperate than in cold-temperate flies (Figure 2). In addition to reduced plasticity, there were instances with no changes between the populations and such with increased temperature-induced expression plasticity in temperate compared to tropical flies (Figure 2). Most notable for the latter are the changes in the plastic response in female *Glut3* expression. In this case, no significant temperature-induced expression plasticity was found for the cold-temperate Swedish sample, whereas both the warm-temperate French as well as the tropical Zambian sample exhibited expression plasticity due to rearing temperature. This plasticity appeared to be further increased in the French sample compared to the Zambian sample with a more than twice the fold-change in expression between 15°C and 28°C (Figure 2, Supplementary Figure S3, Tables S5 and S8).

Changes in expression plasticity between populations were also detected in cases in which temperature-sensitive expression was reversed with a higher expression at 28°C than at 15°C. Whereas the temperature response of *twi* expression in Swedish males and testes consisted of a reduction in plasticity relative to the ancestral value, expression plasticity was increased in the French sample. Reduced plasticity in Swedish flies was largely achieved by downregulating gene expression at 28°C relative to ancestral expression, while the increased French plasticity resulted from downregulation at 15°C (Figures 2 and 3, Supplementary Figure S4, Tables S5, S6, and S9).

The buffering effect on PcG-type temperature-sensitive expression in temperate flies that was observed for the majority of the examined PcG targets was achieved through different changes in expression depending on gene and context. Both upregulation at 28°C and downregulation at 15°C relative to ancestral levels contributed to the reduced plastic response in temperate flies, often both together in a less distinct manner (Figure 3, Supplementary Figures S3–S11, Tables S8–S16). In particular, for whole-fly expression, this lack of clear patterns might be attributed to the averaging of expression across much different tissue and cell types. In other cases, however, the differences out of which reduced plasticity arose were more clear-cut. Lower expression at 15°C relative to ancestral levels, for instance, led to reduced plasticity of *Prat2* expression in ovaries of Swedish and French flies (Supplementary Figure S7 and Table S12), while higher expression at 28°C in ovaries resulted in less plasticity of *Appl* expression in both temperate populations (Supplementary Figure S8 and Table S13). In other cases, an overall higher (e.g., *ninaC* expression in testes of French flies) or lower (e.g., *Glut3* expression in Swedish female flies), more stable expression across temperatures was behind the reduction of temperature-induced expression plasticity in temperate flies (Supplementary Figures S3 and S9, Tables S8 and S14).

## Discussion

Variation in temperature is known to affect epigenetic regulation by the PcG (Fauvarque and Dura 1993). This sensitivity to temperature leads to differences in the transcriptional output of PcG-regulated genes with higher expression levels at lower temperature (Fauvarque and Dura 1993; Chan *et al.* 1994; Zink and Paro 1995; Bantignies *et al.* 2003; Gibert *et al.* 2011; Voigt *et al.* 2015). In this study, we examined PcG target genes in *D.* *melanogaster* for differences in temperature-sensitive expression between populations from different climates and between different fly stages and tissues. We included PcG target genes for which PcG-type temperature-sensitive expression was shown before, though only in whole-fly males and in a different temperature regimen (Zhao *et al.* 2015). However, we also considered genes which displayed no significant temperature-sensitive expression in the aforementioned study as well as those with a temperature response reversed to the one typical observed for PcG-regulation.

PcG-type expression plasticity was observed for nearly all of the studied target genes in at least one of the stages/sexes and tissues examined. Changes in the regulatory machinery are the likely causes of the observed higher expression of PcG target genes at lower temperature. At least though for whole-fly expression, changes in expression between temperatures might also result from differences in the scaling relationships between organs. Variation in rearing temperature affects body and organ size of the fly, however, it has been shown that changes in size due to temperature can be stronger for one organ than the other (Shingleton *et al.* 2009). This alone could, therefore, lead to overall expression changes between flies reared at different temperatures. However, since we also detected higher tissue-specific expression at lower temperatures, and this has often been observed before such as in the classic experiments that demonstrated temperature-sensitive PcG regulation using red-eye color as a marker (Fauvarque and Dura 1993; Chan *et al.* 1994; Zink and Paro 1995; Bantignies *et al.* 2003), regulatory changes are most likely involved in causing increased transcription at lower temperature.

Unexpectedly, nearly none of the PcG-type temperature-sensitive expression was observed in males. In contrast, a large majority of the PcG targets displayed PcG-type temperature-sensitive expression in females, including those genes with reversed temperature-induced expression plasticity in males. Tissue-specific expression analysis, however, revealed that PcG-type temperature-sensitive was also apparent in males. There was only one PcG target gene overlapping in its temperature response between the sexes, which was seven up (*svp*) whose product is as many PcG targets involved in development (Miller *et al.* 2008; Trujillo *et al.* 2016), but also appears to be an important regulator of insulin signaling and lipid metabolism (Musselman *et al.* 2018). Interestingly, the degree of temperature-induced plasticity of *svp* expression varied among flies from different climates with a generally higher overexpression at lower temperatures in tropical than in temperate flies.

This pattern of reduced PcG-type temperature-sensitive expression in temperate compared to tropical ancestral populations was observed for most of the PcG target genes studied here. Similar reductions of temperature-induced expression plasticity in temperate compared to tropical flies were observed before in population samples collected from different locations across different continents (Levine *et al.* 2011; Voigt *et al.* 2015, 2019; Zhao *et al.* 2015). Altogether, this appears to support the idea that temperature-induced expression plasticity of PcG-regulated genes can be detrimental, and therefore is buffered in flies adapted to temperate climates in order to maintain consistent expression levels across temperatures (Levine and Begun 2008). Since PcG proteins are also involved in regenerative processes (Grossniklaus and Paro 2014; Chou *et al.* 2019; Harris *et al.* 2020), an alternative explanation might be that the upregulation of PcG target genes serves to control the damage from stress experienced at lower temperatures. With temperate flies being better adapted to colder conditions (Hoffmann *et al.* 2003; Ayrinhac *et al.* 2004; Svetec *et al.* 2011; Pool *et al.* 2017), they might experience less stress resulting in less damage, and thus less upregulation of PcG target genes at lower temperatures in contrast to tropical flies. However, temperate populations also differed in the occurrence and degree of this buffering with warm-temperate flies showing more often and stronger reduction of plasticity than cold-temperate ones. An explanation for this might be that the range in which temperatures vary in warm-temperate climates encompass cold and hot temperatures, similar to the ones of this study, with greater regularity, whereas in cold-temperate and tropical climates the range is generally restricted to colder and hotter temperatures, respectively.

Although in the majority of cases ancestral overexpression at lower temperatures appeared to be of disadvantage in (warm-)temperate climates, there were also instances in which overexpression at 15°C seemed to be neutral and was roughly the same across populations. Moreover, in some cases temperature-induced plasticity was even further increased in temperate compared to ancestral tropical populations. In the latter, one could assume that such an increase of expression plasticity might have been advantageous and selected for in temperate environments. In the case of female *Glut3* expression, in which ancestral plasticity appeared to be buffered in cold-temperate flies and increased in warm-temperate flies, there is evidence for positive selection acting on the *Glut3* locus in natural populations of *D. melanogaster*. Fabian *et al.* (2012) found significant genetic differentiation in the *Glut3* gene region among three populations along the North American east coast derived from similar climates as those in this study. Significant genetic differentiation, which is indicative of positive directional selection, was observed between temperate and tropical populations and interestingly, also between the warm- and cold-temperate populations. Moreover, another study also observed significant genetic differentiation in the *Glut3* gene region between temperate and tropical populations from Australia (Kolaczkowski *et al.* 2011).

Significant genetic differentiation between temperate and tropical populations was also detected in the gene regions of four other PcG targets of this study (*hh*, *twi*, *gl*, and *lbl*) (Fabian *et al.* 2012). Such genetic variation might be indicative of possible *cis*-regulatory changes responsible for differentiated expression patterns between temperate and tropical populations. The different expression responses to temperature might, however, also result from selection acting on *trans*-regulatory factors such those of the PcG and TrxG. Indeed a subunit of one of the major PcG repressive complexes, *polyhomeotic-proximal* (*ph-p*), was found to be significantly differentiated in its gene region between temperate and tropical flies from the North American cline (Fabian *et al.* 2012) and also between European temperate and African tropical populations (Voigt *et al.* 2015). Latter included the French and Zambian populations of this study. Strong genetic differentiation along the North American cline was also observed in the gene regions of six members of the TrxG, the group of epigenetic regulators that counteract the PcG (Fabian *et al.* 2012).

Testes and ovaries appeared to contribute to the observed temperature-sensitive expression in adults, whereas female midguts did less so for the PcG target genes examined in this study. However, since the majority of the studied target genes exhibited temperature-sensitive expression in whole-fly females, but less than half did so in the examined female tissues, other tissues likely play a role as well. How buffering of temperature-induced expression plasticity in the temperate populations was achieved also differed among genes. Both downregulation at lower temperatures and upregulation at higher temperatures relative to ancestral expression levels contributed to the reduced temperature response in temperate flies. This together with the likely diverse range of tissues in which temperature-sensitive expression is present and buffered, might hint at more factors with smaller effects being responsible for the reduction temperature-sensitivity rather than one *trans*-acting factor with a large effect. However, to further clarify this question more comprehensive studies need to be performed. The present study elucidated how temperature-sensitivity of PcG regulation can vary among PcG target genes, between different stages and tissues, as well as between populations adapted to different climates. Tissue-specific studies encompassing all PcG target genes would be helpful to determine the proportions of target genes with temperature-sensitive expression and those with reduced and increased plasticity in temperate flies. It would be also interesting whether there are particular sets of target genes and tissues more affected by temperature than others, as well as the types of target genes and tissues with changes in temperature-induced expression plasticity between populations adapted to different climates.
